# Validation of CURB-65, CRB-65, NEWS2, qSOFA, and 4C scores for predicting mortality in COVID-19 patients across seven emergency Departments in Colombia

**DOI:** 10.3389/fmed.2026.1738978

**Published:** 2026-02-18

**Authors:** Sandra Liliana Valderrama-Beltrán, Juliana Cuervo-Rojas, Martín Rondón, Samuel Martinez-Vernaza, Ilich Herbert De La Hoz Siegler, Alfonso J. Rodriguez-Morales, Alejandra Cañas-Arboleda, Oscar Muñoz, Mónica Padilla, María Lucía Mesa-Rubio, José Antonio Rojas, Juan Sebastián Bravo Ojeda, Jaime Villa, Julio Alberto Chacón Sarmiento, Sandra Patiño, Roberto Tarud Ayub, Claudia Aristizábal, Paola Rengifo, Ginna Tambini, Silvia Bertagnolio, Janet Diaz, Ludovic Reveiz, Carlos Álvarez-Moreno

**Affiliations:** 1 PhD Program in Clinical Epidemiology, Department of Clinical Epidemiology and Biostatistics, Faculty of Medicine, Pontificia Universidad Javeriana, Bogotá, Colombia; 2 Division of Infectious Diseases, Department of Internal Medicine, Infectious Diseases Research Group, Hospital Universitario San Ignacio, Bogotá, Colombia; 3 Department of Clinical Epidemiology and Biostatistics, Faculty of Medicine, Pontificia Universidad Javeriana, Bogotá, Colombia; 4 Department of Internal Medicine, Faculty of Medicine, Pontificia Universidad Javeriana, Bogotá, Colombia; 5 Department of Internal Medicine, Hospital Universitario San Ignacio, Bogotá, Colombia; 6 Clinica Colsanitas, Bogotá, Colombia; 7 Faculty of Health Sciences, Universidad Cientifica del Sur, Lima, Peru; 8 Grupo de Investigación Biomedicina, Faculty of Medicine, Fundación Universitaria Autónoma de las Américas-Institución Universitaria Visión de las Américas, Pereira, Colombia; 9 Pan American Health Organization, Bogotá, Colombia; 10 Universidad de Los Andes, Bogotá, Colombia; 11 Fundación Universitaria Sanitas, Clínica Universitaria Colombia, Bogotá, Colombia; 12 Clínica Infantil Santa María del Lago, Bogotá, Colombia; 13 Clínica el Carmen, Barranquilla, Colombia; 14 Clínica Reina Sofia, Bogotá, Colombia; 15 Clínica Sebastián de Belalcázar, Cali, Colombia; 16 Clínica Iberoamérica, Barranquilla, Colombia; 17 Unidad de Investigación Clínica, Fundación Universitaria Sanitas, Bogotá, Colombia; 18 Department of Evidence and Intelligence for Action in Health (EIH), Pan American Health Organization, Washington, DC, United States; 19 Clínica Colsanitas and Facultad de Medicina, Universidad Nacional de Colombia, Bogotá, Colombia

**Keywords:** COVID-19, emergency medicine, mortality prediction, prognostic models, risk assessment

## Abstract

**Background:**

Accurate risk stratification is essential for guiding hospitalization decisions in COVID-19. We evaluated the performance of CURB-65, CRB-65, NEWS2, qSOFA, and the 4C Mortality Score in predicting 30-day mortality among patients presenting to the emergency department with COVID-19.

**Methods:**

We conducted an external validation study using an ambispective cohort of COVID-19 patients who presented to emergency departments of seven high-complexity hospitals in Colombia between March 2020 and September 2021. We assessed discrimination using the area under the receiver operating characteristic curve (AUC) and calibration using the GiViTI belt, the observed-to-expected (O/E) ratio, and the calibration intercept and slope. Decision curve analysis and net benefit were used to evaluate clinical utility. The 4C model underwent logistic recalibration.

**Results:**

Among 7,973 patients included, 30-day mortality was 11.3%. The 4C model showed the highest discrimination (AUC 0.71, 95% CI 0.70–0.73) and clinical utility, but poor calibration. NEWS2, CURB-65, CRB-65, and qSOFA performed poorly across all performance metrics. After recalibration, the 4C model achieved an O/E ratio of 1 and showed a modest improvement in discrimination. Decision curve analysis confirmed its utility for guiding hospitalization decisions at a ≥4% mortality risk threshold.

**Conclusion:**

The 4C Mortality Score outperformed other models in predicting COVID-19 mortality. Its use in emergency settings alongside clinical judgment can enhance risk stratification, guide hospitalization decisions, and optimize resource allocation. Recalibration and decision analysis are essential for its clinical applicability. Further validation with contemporary data is essential to ensure its transportability across epidemiological settings.

## 1 Introduction

Although the COVID-19 pandemic has officially ended, SARS-CoV-2 remains a global public health concern (1). It is hazardous for immunosuppressed individuals and those with comorbidities such as diabetes, cardiovascular disease, chronic kidney disease, and obesity, who face higher risks of severe complications and mortality (2, 3). Despite widespread vaccination, COVID-19 has become endemic, necessitating practical tools to identify patients at high risk of unfavorable outcomes (4).

Given the broad clinical spectrum, accurate triage in emergency settings is crucial. Predictive models assist clinicians in determining which patients require outpatient management versus hospitalization, optimizing resource allocation, and improving outcomes (5, 6). Over 50 prognostic models have been developed or validated for COVID-19 (6, 7), among them CURB-65, NEWS2, qSOFA, and the 4C mortality score, which have been widely studied for assessing prognosis and guiding hospitalization decisions (6, 7).

These models are valued for their simplicity, minimal laboratory requirements, and clinical applicability in conditions such as pneumonia and sepsis (8). The 4C model, developed explicitly for COVID-19, demonstrated adequate validity, discrimination, and calibration in predicting mortality (9). However, despite numerous prognostic models, external validation studies remain limited, and few have comprehensively evaluated calibration performance and clinical utility—particularly through decision curve analysis—to inform real-world clinical decision-making (6, 10). Given the potential for model overfitting and changes in disease severity, circulating variants, and healthcare delivery over time, external validation is essential to assess whether a prediction model maintains its accuracy and clinical utility across diverse populations, settings, and epidemiological conditions. This study aimed to validate the predictive performance of CURB-65, CRB-65, NEWS-2, qSOFA, and 4C in patients with laboratory-confirmed COVID-19 for predicting 30-day mortality following an emergency department visit, using data from the Colombian cohort of the World Health Organization’s Global Clinical Platform (11).

## 2 Methods

### 2.1 Study design, setting, and participants

This study validates prognostic models using data from an ambispective cohort of COVID-19 patients within the WHO Global Clinical Platform (11), to which Colombia contributed anonymized, individual-level data. The platform uses standardized ISARIC case report forms and a predefined analytic framework, with harmonized variable domains including demographics, baseline comorbidities, clinical presentation at admission, laboratory parameters, treatments, and clinical outcomes (11).

The cohort included patients aged 18 years or older who presented to the emergency department with suspected SARS-CoV-2 infection, later confirmed by RT-PCR or antigen testing, between March 2020 and September 2021. Data were collected from seven high-complexity hospitals: four in Bogotá, two in Barranquilla, and one in Cali.

Patients were included if they had confirmed SARS-CoV-2 infection and at least one COVID-19-related symptom (see Supplementary material M1). Exclusion criteria included transfer to another institution within 24 h or teleconsultation. To avoid duplicate observations, only the first emergency department visit per patient during the study period was included in the analysis. Cohort entry began at the time of the first emergency department consultation, and all patients were followed for up to 30 days after admission.

Model validation followed the TRIPOD (transparent reporting of a multivariable prediction model for individual prediction or diagnosis) guidelines (12, 13).

### 2.2 Variables

The variables were those originally included in each model. They encompassed demographic variables, comorbidities (modified Charlson Comorbidity Index) (14), vital signs at admission, clinical parameters (use of supplemental oxygen, and presence of confusion), and admission laboratory tests (urea, C-reactive protein). The recorded comorbidities were chronic cardiac disease, chronic respiratory disease (excluding asthma), chronic renal disease (estimated glomerular filtration rate ≤30), mild to severe liver disease, dementia, chronic neurological conditions, connective tissue disease, diabetes mellitus, HIV/AIDS, malignancy, and clinician-defined obesity.

The primary outcome was 30-day mortality, defined as death from any cause within 30 days from the emergency department visit. For discharged patients, survival status was confirmed via the Registro Único de Afiliados (RUAF) of the Colombian Ministry of Health (15). A blinded investigator verified death certificates. Additional data on hospitalization, intensive care unit (ICU) admission, and hospital stay length were also collected.

All seven participating hospitals used electronic health record (EHR) systems, which allowed standardized extraction of clinical variables using definitions aligned with the WHO/ISARIC case report forms (11, 16). Data entry followed the standardized procedures of the WHO Global Clinical Platform and was performed either through direct entry into the platform or via upload of a harmonized WHO Excel template. Laboratory data were transferred electronically from institutional laboratory information systems to the ISARIC database in all centers, ensuring uniform units of measurement and minimizing transcription errors. Data collection was performed by trained physician-investigators at each site. To ensure data quality and completeness, 10% of the sample was independently reviewed in duplicate. In addition, two investigators conducted monthly reviews of the Colombian National Public Health Surveillance System to verify consecutive patient inclusion and data accuracy.

### 2.3 Sample size

Sample size calculation followed the Riley et al. methodology (17), using the 4C model (*n* = 20 parameters, C-statistic 0.78) and an estimated 11.2% mortality rate observed in a previous study based on the Colombian WHO Global Clinical Platform cohort (18). The minimum required sample size was 1,624 patients with 82 mortality events. Given our cohort of 7,973 patients and 901 deaths, the sample was sufficient for model validation.

Because the 4C model includes the largest number of predictors and represents the most statistically demanding validation scenario, meeting its sample size requirements also ensured adequate sample size for the validation of CURB-65, NEWS2, and qSOFA models.

### 2.4 Statistical analysis

Exploratory data analysis identified outliers, entry errors, and missing values, and adjustments were made as needed. No missing values were found in demographic or clinical variables. However, C-reactive protein and urea were missing in 2.0% (*n* = 159) and 2.7% (*n* = 216) of patients, respectively. The only model that incorporated both variables was the 4C, with 4.6% (*n* = 369) missing data. Since this percentage was below 5%, a complete-case analysis was conducted. There was no missing data for the outcome variable.

A descriptive analysis was conducted, summarizing continuous variables as medians and interquartile ranges (IQRs), and categorical variables as absolute and relative frequencies. The Kaplan–Meier method was used to estimate overall survival, with comparisons across predefined risk groups performed using log-rank tests for each model. Administrative censoring was applied at 30 days post-admission.

For model validation, mortality predictions were generated using CURB-65, CRB-65, NEWS2, qSOFA, and 4C models (see Supplementary material M2), applying the original scoring criteria (9, 19–21). Discrimination was assessed using the receiver operating characteristic (ROC) curves and the area under the curve (AUC) with a 95% confidence interval (95% CI), with AUC > 0.7 considered acceptable (13).

Calibration was evaluated using the GiViTI calibration belt. This method incorporates a confidence band to quantify prediction uncertainty and employs a likelihood ratio test, and to capture non-linear deviations from perfect calibration (22). Additionally, observed-to-expected (predicted) events were analyzed by risk group to provide a detailed performance assessment.

In addition, for the 4C and NEWS2 models, we conducted analyses to evaluate potential changes in model performance across key phases of the pandemic in Colombia. Discrimination and calibration were assessed separately for two relevant time periods: (a) before and after the implementation of corticosteroid therapy in routine clinical practice (July 2020) (23), and (b) before and after the launch of the national COVID-19 vaccination program in Colombia (April 2021).

For the 4C model, which provided intercept and regression coefficients (9). We calculated additional overall performance measures such as the Cox-Snell R-squared, Brier Score, and calibration metrics such as the calibration intercept and calibration slope.

Despite its good discriminatory performance, the 4C model exhibited poor calibration. As initial recalibration-in-the-large (adjusting only the intercept) was insufficient to correct this miscalibration. Therefore, logistic recalibration, involving adjustments to both the intercept and slope, was performed, resulting in improved discrimination and calibration. The choice of recalibration method was supported by a likelihood ratio test, which identified this approach as the most conservative and statistically appropriate (24, 25).

Finally, to assess the clinical utility of the 4C model, we conducted decision curve analysis (DCA) and evaluated net benefit, which balances intervention benefits against potential harms. To determine an appropriate risk threshold for death to guide hospitalization decisions for COVID-19, we selected a risk threshold range between 2 and 10% based on expert clinical consultation and previous prediction models (9, 19–21). The recalibrated 4C model was compared to the original model, and the strategies of hospitalizing all or none (10, 26).

Data analysis was performed in R v4.1 using the packages pmsampsize, predRupdate, pROC, givitiR, rms, and rmda.

### 2.5 Ethical aspects

The study was approved by the Research and Ethics Committee of HUSI and Pontificia Universidad Javeriana (FM-CIEI-0686-21). The Institutional Review Board (IRB) approved each participating institution’s participation in the WHO Global Clinical Platform (11), whose data serve as the basis for this study. Informed consent was waived due to the retrospective design. Data were anonymized, with access restricted to designated investigators.

## 3 Results

### 3.1 Population characteristics

A total of 7,973 out of 9,050 patients from the Colombian cohort of the WHO Global Clinical Platform for COVID-19 met the inclusion criteria (see Supplementary material F1). In the study population, 58.3% were men, and the median age was 60.7 years (IQR: 49–80). At 30 days, the median age was 59.6 years (IQR: 48.3–80.1) and 70.6 years (IQR: 60.1–81.6) among survivors and non-survivors, respectively.

The most frequent underlying condition was hypertension (29.5%, *n* = 2,353), followed by obesity (27.6%, *n* = 2,202) and diabetes mellitus (16.9%, *n* = 1,346). Among non-survivors, having two or more comorbidities was common (39.3%, *n* = 354) (see Table 1). Overall, 95.7% (*n* = 7,627) of patients required hospitalization for more than 24 h, whereas 13.4% (*n* = 1,072) were admitted to the ICU. The median hospital stay was 6 days (IQR: 3.2–11.4).

**Table 1:** Characteristics of a cohort of patients with SARS-CoV-2 infection presenting to seven high-complexity emergency departments in Colombia between March 2020 and September 2021.

Variable	Total (*N* = 7,973)^1^	Survivor (*N* = 7,072)^1^	Non-survivor (*N* = 901)^1^
Age (years), *n* (%)			
<50	2,118 (26.6%)	2007 (28.4%)	111 (12.3%)
50–59	1716 (21.5%)	1,595 (22.6%)	121 (13.4%)
60–69	1865 (23.4%)	1,663 (23.5%)	202 (22.4%)
70–79	1,286 (16.1%)	1,056 (14.9%)	230 (25.5%)
≥80	988 (12.4%)	751 (10.6%)	237 (26.3%)
Sex at birth, *n* (%)			
Male	4,652 (58.3%)	4,102 (58.0%)	550 (61.0%)
Female	3,321 (41.7%)	2,970 (42.0%)	351 (39.0%)
Temperature (°C)^2^, median (IQR^3^)	36.5 (36.0–37.0)	36.5 (35.5–37.5)	36.5 (35.5–37.5)
Temperature (°C)^2^, *n* (%)			
≤35	91 (1.1%)	73 (1.0%)	18 (2.0%)
35.1–36	2007 (25.2%)	1777 (25.1%)	230 (25.5%)
36.1–38	5,300 (66.5%)	4,712 (66.6%)	588 (65.3%)
38.1–39	482 (6.0%)	429 (6.1%)	53 (5.9%)
>39	93 (1.2%)	81 (1.1%)	12 (1.3%)
Heart rate^4^, median (IQR^3^)	85 (76–99)	85 (61–109)	86 (63–109)
Heart rate^4^, *n* (%)			
≤40	11 (0.1%)	3 (0.0%)	8 (0.9%)
41–50	48 (0.6%)	44 (0.6%)	4 (0.4%)
51–90	4,915 (61.6%)	4,364 (61.7%)	551 (61.2%)
91–110	2012 (25.2%)	1796 (25.4%)	216 (24.0%)
111–130	861 (10.8%)	770 (10.9%)	91 (10.1%)
>130	126 (1.6%)	95 (1.3%)	31 (3.4%)
Respiratory rate^5^, median (IQR^3^)	20 (18–22)	20 (16–24)	21 (15–27)
Respiratory rate^5^, *n* (%)			
<22	5,288 (66.3%)	4,825 (68.2%)	463 (51.4%)
≥22	2,685 (33.7%)	2,247 (31.8%)	438 (48.6%)
Systolic blood pressure^6^, median (IQR^3^)	123 (112–135)	123 (100–146)	124 (98–152)
Systolic blood pressure^6^, *n* (%)			
<90	204 (2.6%)	149 (2.1%)	55 (6.1%)
≥90	7,769 (97.4%)	6,923 (97.9%)	846 (93.9%)
Diastolic blood pressure^6^, median (IQR^3^)	75 (69–75)	75 (62–88)	73 (57–89)
Diastolic blood pressure^6^, *n* (%)			
≤60	1,002 (12.6%)	821 (11.6%)	181 (20.1%)
>60	6,971 (87.4%)	6,251 (88.4%)	720 (79.9%)
Glasgow Coma Scale^7^, median (IQR^3^)	15 (15–15)	15 (15–15)	15 (14–16)
Glasgow Coma Scale^7^, *n* (%)			
<15	1,500 (18.8%)	1,251 (17.7%)	249 (27.6%)
15	6,473 (81.2%)	5,821 (82.3%)	652 (72.4%)
Oxygen saturation^8^, median (IQR^3^)	92 (88–94)	92 (89–95)	90 (85–94)
Oxygen saturation^8^, *n* (%)			
≤91%	3,852 (48.3%)	3,330 (47.1%)	522 (57.9%)
92–93%	1,502 (18.8%)	1,353 (19.1%)	149 (16.5%)
94–95%	146 (15.6%)	1,129 (16.0%)	117 (13.0%)
≥96%	1,373 (17.2%)	1,260 (17.8%)	113 (12.5%)
Confusion^9^, *n* (%)	1,500 (18.8%)	1,251 (17.7%)	249 (27.6%)
Underlying conditions^10^, *n* (%)			
Hypertension	2,353 (29.5%)	2008 (28.4%)	345 (38.3%)
Chronic cardiac disease	280 (3.5%)	226 (3.2%)	54 (6.0%)
Diabetes mellitus	1,346 (16.9%)	1,145 (16.2%)	201 (22.3%)
Chronic renal disease	1,284 (16.1%)	1,067 (15.1%)	217 (24.1%)
Clinician-defined obesity	2,202 (27.6%)	1974 (27.9%)	228 (25.3%)
Chronic respiratory disease	1,085 (13.6%)	923 (13.1%)	162 (18.0%)
Asthma	118 (1.5%)	101 (1.4%)	17 (1.9%)
Connective tissue disease	171 (2.1%)	143 (2.0%)	28 (3.1%)
Liver disease	259 (3.2%)	220 (3.1%)	39 (4.3%)
Chronic neurological conditions	150 (1.9%)	112 (1.6%)	38 (4.2%)
Malignancy	1,125 (14.1%)	941 (13.3%)	184 (20.4%)
HIV/AIDS^11^	212 (2.7%)	198 (2.8%)	14 (1.6%)
Underlying conditions number, *n* (%)			
0	4,593 (57.6%)	4,163 (58.9%)	430 (47.7%)
1	952 (11.9%)	835 (11.8%)	117 (13.0%)
2 or more	2,428 (30.5%)	2074 (29.3%)	354 (39.3%)
C-reactive protein, mg/L (*n* = 7,757), median (IQR^3^)	69.2 (20.4–146.2)	66.5 (37.6–95.3)	102.1 (57.5–146.6)
C-reactive protein (*n* = 7,757), *n* (%)			
<50 mg/L	3,161 (40.8%)	2,905 (42.0%)	256 (30.2%)
50–99 mg/L	1,692 (21.8%)	1,529 (22.1%)	163 (19.2%)
≥100 mg/L	2,904 (37.4%)	2,475 (35.8%)	429 (50.6%)
Urea, mmol/L (*n* = 7,816), median (IQR^3^)	6.8 (5.5–8.2)	6.7 (5.5–8.0)	7.7 (5.5–9.8)
Urea (*n* = 7,816), *n* (%)			
≤7 mmol/L	4,073 (52.1%)	3,731 (53.8%)	342 (38.7%)
>7 mmol/L	3,743 (47.9%)	3,201 (46.2%)	542 (61.3%)
Hospitalization, *n* (%)	7,627 (95.7%)	6,771 (95.7%)	856 (95.0%)
Dexamethasone use^12^, *n* (%)	6,933 (87.0%)	6,173 (87.3%)	760 (84.3%)
ICU admission^13^, *n* (%)	1,072 (13.4%)	675 (9.5%)	397 (44.1%)
Length of hospital stay, median (IQR^3^)	6.0 (3.2–11,4)	5.6 (2.0–9.2)	11.0 (4.8–17)

1. Column-based percentages. 2. Body temperature in degrees Celsius (°C) at admission. 3. IQR: interquartile range. 4. Heart rate in beats per minute (bpm) at admission. 5. Respiratory rate in breaths per minute (bpm) at admission. 6. Blood pressure in millimeters of mercury (mmHg) at admission. 7. Glasgow Coma Scale score at admission. 8. Oxygen saturation at admission. 9. Confusion at admission. 10. Patients may have multiple underlying conditions; thus, categories are not mutually exclusive. 11. Human immunodeficiency virus (HIV) infection. 12. Dexamethasone use was defined according to the RECOVERY trial protocol for COVID-19, which recommends a dose of 6 mg once daily for up to 10 days in hospitalized patients requiring oxygen therapy or an equivalent dose of another corticosteroid (34). 13. ICU: Intensive care unit.

At 30 days post-admission to the emergency department, the cumulative incidence of death was 11.3% (*n* = 901). The Kaplan–Meier survival curves, stratified by NEWS2, CURB-65, qSOFA, and 4C score categories, demonstrate significant differences in survival probability across risk groups (log-rank test, *p* < 0.0001). For all models, patients in the highest-risk category experience a decline in survival within the first days of follow-up. In contrast, those in the lowest-risk category maintain high survival probabilities over time (see Supplementary material F2).

### 3.2 Performance of prognostic models

**Figure 1:**
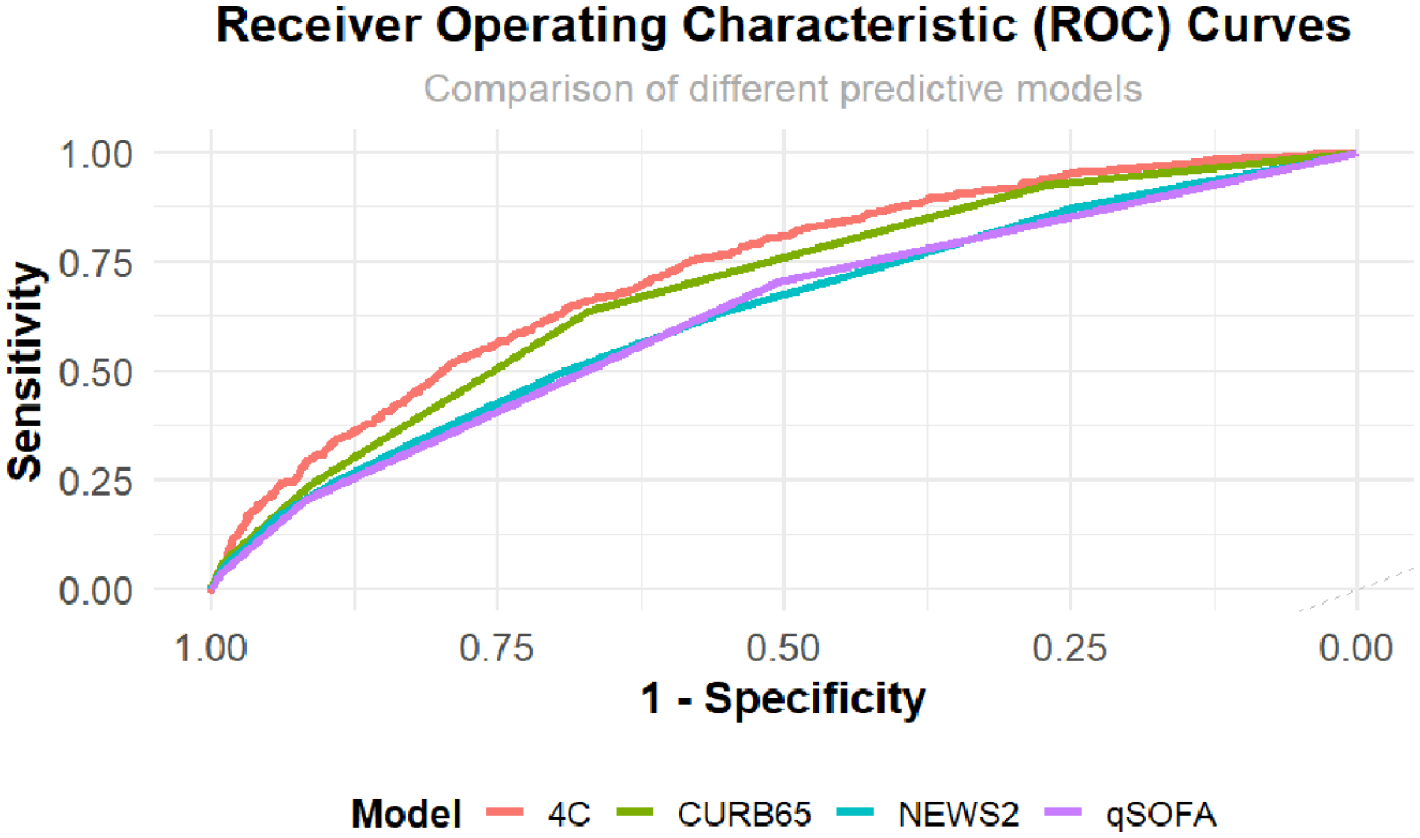
Receiver operating characteristic (ROC) curves of CURB-65, NEWS2, qSOFA, and 4C for predicting 30-day mortality following emergency department admission in a cohort of patients with SARS-CoV-2 infection across seven high-complexity emergency departments in Colombia (March 2020 – September 2021). NEWS2: National Early Warning Score; qSOFA: quick Sequential [Sepsis-related] Organ Failure Assessment; 4C: Coronavirus Clinical Characterization Consortium Mortality Score.

The discriminative performance of the models was assessed using the AUC, yielding values of 0.69 (95% CI: 0.67–0.70) for CURB-65, 0.67 (95% CI: 0.66–0.69) for CRB-65, 0.63 (95% CI: 0.61–0.65) for NEWS2, 0.62 (95% CI: 0.61–0.64) for qSOFA, and 0.71 (95% CI: 0.70–0.73) for the 4C mortality score. Figure 1 presents the ROC curves for the evaluated models.

**Figure 2:**
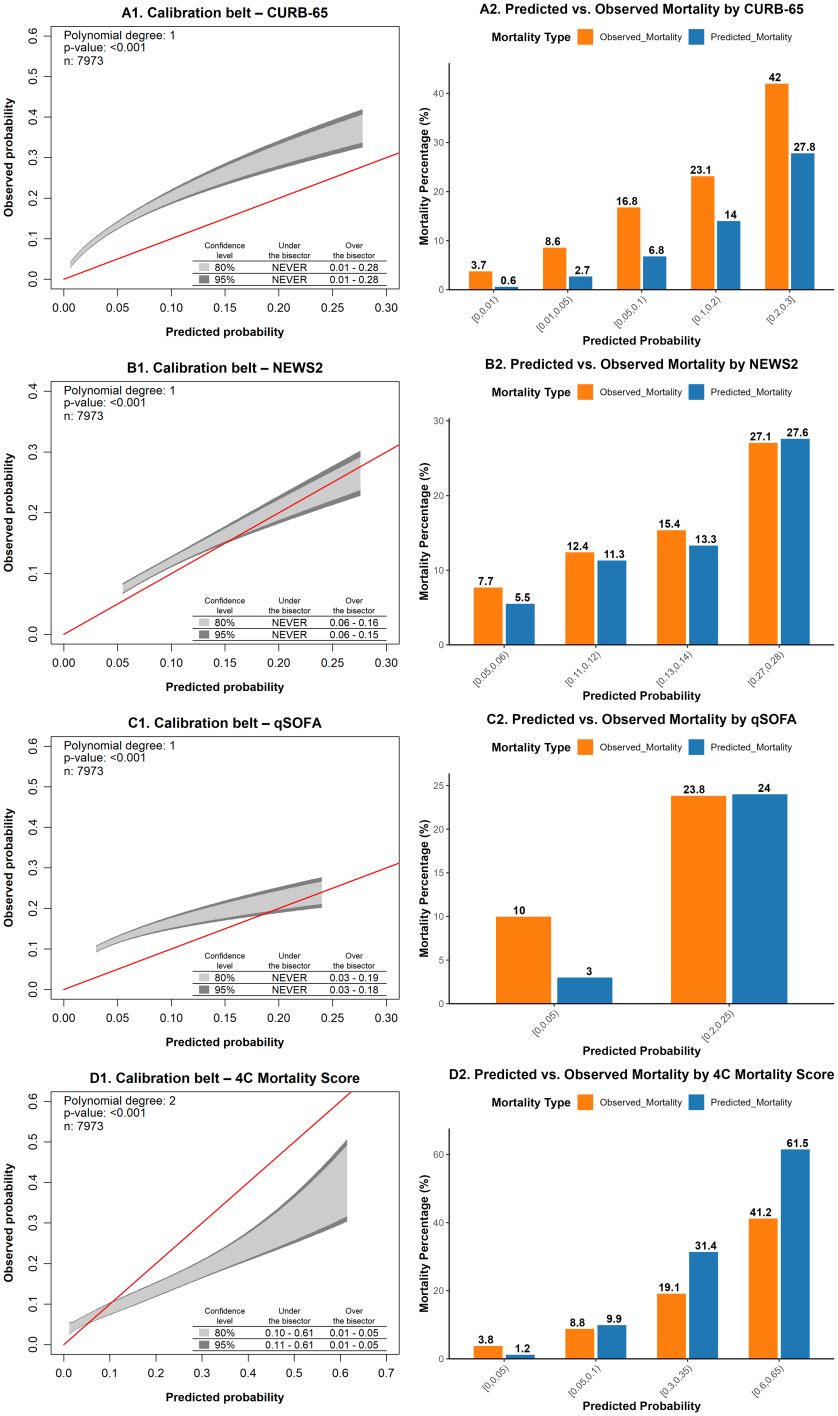
The left side displays calibration belt plots, while the right side presents observed-to-expected (predicted) event plots stratified by predefined risk groups for CURB-65 **(A1,B1)**, NEWS2 **(A2,B2)**, qSOFA **(A3,B3)**, and 4C **(A4,C4)**. These models were used to predict 30-day mortality after admission to the emergency department in a cohort of patients with SARS-CoV-2 infection across seven high-complexity emergency departments in Colombia (March 2020 – September 2021). NEWS2: National Early Warning Score; qSOFA: quick Sequential [Sepsis-related] Organ Failure Assessment; 4C: Coronavirus Clinical Characterization Consortium mortality score.

In terms of calibration, the GiViTI calibration belt analysis indicated that none of the models achieved adequate calibration (Figure 2). Additionally, the likelihood ratio test (*p* < 0.05) rejected the null hypothesis of no differences between predicted and observed probabilities for all models.

An observed-to-expected event plot stratified by predefined risk groups was also generated (Figure 2). The results showed that the CURB-65 and CRB-65 models underestimated mortality risk across all predefined risk groups. The NEWS2 model underestimated mortality risk in the low-to-intermediate risk groups. The qSOFA model underestimated the risk in the low-risk group. Similarly, the 4C model underestimated mortality risk in the lower-risk groups and consistently overestimated it in the remaining groups. Detailed information on the comparison of observed and predicted event probabilities for all models is available in Supplementary Table 1R.

When comparing the performance of the 4C and NEWS2 models before and after the introduction of corticosteroid therapy, or before and after the implementation of COVID-19 vaccination in Colombia, we did not observe clinically meaningful differences in overall discriminative ability. For the NEWS2, no significant differences in calibration were found between the periods. However, the 4C model did show better calibration during the period prior to than during the period following the introduction of corticosteroids. Once there was an effective therapy, the model overestimated the risk of death, especially among those at high risk. Detailed performance metrics for each period are presented in Supplementary Figures F3–F6.

The overall performance of the 4C model is presented in Table 2. Given that the 4C model showed the best discrimination among the evaluated models, we recalibrated it and reassessed its performance.

**Table 2:** Regression coefficients and predictive performance of the original and recalibrated 4C prediction models for 30-day mortality after emergency department admission in patients with SARS-CoV-2 infection.

	Original 4C mortality model^1^	Recalibrated 4C mortality model^2^
Model coefficients		
Intercept	−4.203	−4.047
Age (years)		
<50 (Ref)^3^	–	–
50–59	0.687	0.536
60–69	1.337	1.044
70–79	1.842	1.438
≥80	2.252	1.758
Sex at birth		
Female (Ref)^3^	–	–
Male	0.172	0.134
Number of comorbidities		
0 (Ref)^3^	–	–
1	0.3	0.234
≥2	0.532	0.415
Respiratory rate^4^		
<20 (Ref)^3^	–	–
20–29	0.232	0.181
≥30	0.649	0.507
Peripheral oxygen saturation^5^		
≥92% (Ref)^3^	–	–
<92%	0.577	0.450
Glasgow coma scale <15		
15 (Ref)^3^	–	–
<15	0.558	0.436
Urea (mmol/L)		
<7 (Ref)^3^	–	–
7–14	0.439	0.343
>14	1.011	0.789
C-reactive protein (mg/L)		
<50 (Ref)^3^	–	–
50–99	0.363	0.283
≥100	0.747	0.578
Performance measures^6^		
Calibration (95% CI)^7^		
Observed/expected ratio	0.57 (0.54–0.61)	1.00 (0.94–1.07)
Calibration intercept	−0.77 (−0.85–0.70)	0.00 (−0.07–0.07)
Calibration slope	0.77 (0.70–0.84)	1.00 (0.91–1.09)
Discrimination (95% CI)^7^		
C-statistic (AUROC)^8^	0.71 (0.70–0.73)	0.73 (0.71–0.75)
Overall model fit		
Nagelkerke *R*^2^	0.06	0.23
Cox-Snell *R*^2^	0.03	0.16
Brier Score (95% CI)^7^	0.16 (0.15–0.16)	0.09 (0.08–0.09)

1. The coefficients of the original model correspond to those published by the developers of the 4C model in Knight SR et al. (9). 2. The coefficients of the recalibrated model are based on logistic recalibration. 3. Ref: reference category in the regression model. 4. Respiratory rate (breaths/min). 5. Oxygen saturation on air room (%). 6. Evaluation of the performance in predicting 30-day mortality after admission to the emergency department in a cohort of patients with SARS-CoV-2 infection across seven high-complexity emergency departments in Colombia (March 2020 – September 2021). 7. 95% CI: 95% confidence interval. 8. AUROC: area under the receiver operating characteristic curve.

### 3.3 Recalibrated 4C model

The recalibrated 4C model is of the form: New model = intercept_new_ (−1.049) + overall slope (0.781)* original 4C model logit. This was used to adjust the coefficients of all variables in the logistic model. The original and recalibrated coefficients, along with the performance of the 4C Mortality Score, are presented in Table 2. An online calculator based on the recalibrated model is available at: https://github.com/Mars1971/Recalibrated-4C-Mortality-Score-for-COVID-19-in-Colombia.

The updated model achieved an observed-to-expected event ratio of 1.00 (95% CI, 0.94–1.07), an intercept of 0 (95% CI, −0.07 to 0.07), and a calibration slope of 1 (95% CI, 0.91–1.09) (Table 2). Calibration was also confirmed via a calibration belt plot, and a non-significant likelihood ratio test (*p* = 0.644), indicating no meaningful difference between observed and predicted events (Supplementary Figures F7–F9). Discrimination ability slightly improved (DeLong’s test <0.001), as did overall model performance.

### 3.4 Clinical utility

**Figure 3:**
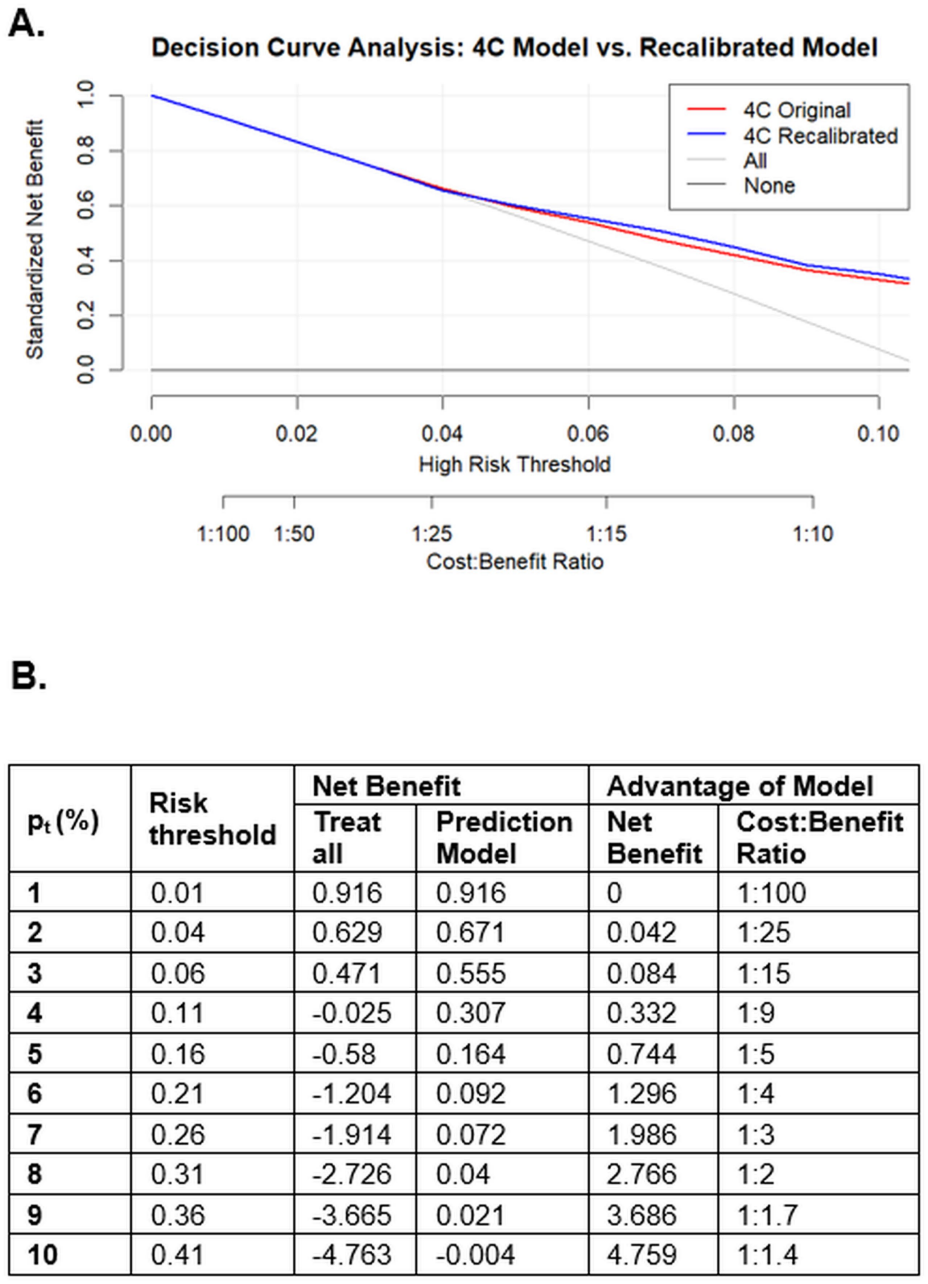
Decision curve analysis **(A)** and net benefit table **(B)** comparing the original and recalibrated 4C mortality scores with both the “all” and “none” hospitalization approaches in a cohort of patients with SARS-CoV-2 infection across seven high-complexity emergency departments in Colombia (March 2020 – September 2021). In the decision curve analysis **(A)**, the *x*-axis represents the selected probability of death threshold for deciding hospitalization, and its corresponding cost–benefit ratio, while the *y*-axis displays the standardized net benefit.

The original and recalibrated 4C models demonstrated a net benefit for guiding hospitalization decisions at a mortality risk threshold of approximately 4% and maintained this benefit across the proposed threshold range. At a 4% mortality risk threshold, the cost–benefit ratio was 1:25, indicating that accepting approximately 25 potentially unnecessary hospital admissions may be justified to avoid missing one patient at high risk of death (Figure 3). A detailed table presenting net benefit values across different mortality risk thresholds is provided in Figure 3. The curves with confidence intervals are shown in the Supplementary Figure F10.

## 4 Discussion

We conducted a comprehensive evaluation of five models used in clinical practice to predict COVID-19 mortality. Among these, the 4C Mortality Score demonstrated the best overall performance, with acceptable discriminative ability, adequate clinical utility, and initially poor calibration, especially after the introduction of corticosteroid therapy. In contrast, in our population, CURB-65, CRB-65, NEWS2, and qSOFA performed poorly in both discrimination and calibration, limiting their clinical usefulness in our setting.

In our cohort, the 4C model outperformed the other evaluated models, exhibiting the highest discriminative ability, consistent with findings from other validation studies (27–31). However, our validation showed a slightly lower AUC than the original study (9).

Regarding calibration, we observed an overestimation of mortality risk at intermediate and high scores and an underestimation at lower scores—a pattern also reported in previous studies (32, 33). These differences may be explained by temporal changes in clinical management and population-level risk over the course of the pandemic. The 4C model was derived during the early pandemic, a period characterized by higher baseline mortality and limited availability of effective therapies, before the routine use of corticosteroids in hypoxemic patients and the implementation of large-scale vaccination programs (34, 35).

In addition to corticosteroid use, other time-varying, treatment-related factors may have contributed to shifts in observed mortality risk, including evolving oxygen therapy protocols, selective use of antivirals, changes in ICU capacity and resource availability during regional surges, and the circulation of different SARS-CoV-2 variants. This is further supported by our stratified analysis of model performance before and after the introduction of corticosteroid therapy, in which the 4C model demonstrated better calibration during the period prior to its introduction (see Supplementary material F6), underscoring the impact of changing treatment contexts on model performance.

Despite the calibration being suboptimal, the 4C model remains a valuable tool, as it effectively stratifies patients based on disease severity, enabling the identification of those at higher or lower risk of mortality (7). To address this issue, we recalibrated the model for our cohort, which is mainly composed of patients receiving corticosteroid therapy, achieving near-perfect calibration while preserving discriminative ability. The recalibrated model demonstrated clinical utility in guiding in-hospital monitorization or hospitalization decisions, particularly at a mortality probability threshold of 4% or higher.

Our validation showed that the NEWS-2 model exhibits inadequate discriminative ability and underestimated mortality risk at low scores, as observed in multiple studies (9, 28, 30, 36). We believe these performance limitations stem from the exclusion of key variables such as age, comorbidities, and critical laboratory parameters—a design choice intended to facilitate rapid bedside assessment—all of which have been previously identified as significant risk factors for unfavorable outcomes (3, 37, 38).

We found that both CURB-65 and CRB-65 consistently underestimated mortality risk across all score values, a limitation previously reported in two extensive validation studies (9, 28). These tools, developed initially for community-acquired pneumonia (CAP), may not be suitable for COVID-19-related CAP, which often presents with silent hypoxemia and may rapidly progress to acute respiratory distress syndrome (39, 40). In line with current American Thoracic Society and the Infectious Diseases Society of America ATS/IDSA guidelines (41), CURB-65 and CRB-65 are no longer recommended for guiding hospitalization decisions or level of care in CAP.

Similarly, our data suggest that qSOFA may underestimate mortality risk, particularly at low scores that classify patients as not at high risk of in-hospital mortality. We observed that nearly 10% of patients who died had a qSOFA score of 0 or 1 upon admission. This limited predictive accuracy has also been reported in other validation studies (7, 9, 40, 42) and may be attributed to the phenomenon of silent hypoxemia, as previously described (39, 40). Therefore, we consider that qSOFA should not be used as a triage tool in patients with suspected COVID-19-related CAP.

To our knowledge, this study represents one of the largest multicenter external validation efforts of prognostic models for COVID-19 mortality conducted in Latin America to date. Although COVID-19 mortality has declined, the disease has become endemic, making the identification of optimal triage strategies essential for clinical decision-making in the emergency setting, as these patients will continue to present for care.

To improve the calibration of the 4C model, it was necessary to adjust both the intercept and the calibration slope. This was possible because the 4C model was developed with methodological rigor, and its original intercept and beta coefficients were fully reported (9, 10). In contrast, recalibration of the NEWS2, CURB-65, and qSOFA models is limited, as their coefficients are not publicly available.

Furthermore, recalibration is a recommended strategy to enhance model performance and clinical usefulness across diverse settings (32). In our study, this process optimized the predictive accuracy of the 4C model in a high-complexity hospital care context in Latin America. To support implementation in similar settings, we developed an online calculator incorporating the recalibrated 4C Mortality Score: https://github.com/Mars1971/Recalibrated-4C-Mortality-Score-for-COVID-19-in-Colombia.

Nonetheless, our study has certain limitations. First, the validation was conducted during a phase of the pandemic when most hospitalized patients had not yet received the COVID-19 vaccination, and before the widespread circulation of the Omicron variant (35). These factors may have influenced the model’s performance and limited the generalizability of our findings to current clinical settings.

However, our results are consistent with more recent validation studies of the 4C model, which have demonstrated stable predictive performance and support its use in clinical practice (6, 31), further reinforcing the robustness of our findings. Moreover, many resource-limited settings continue to face limited access to vaccination and effective therapies, underscoring the continued relevance of our findings in such contexts.

To further strengthen the model’s clinical applicability, temporal validation and, if necessary, recalibration using contemporary data are essential, especially in the context of evolving epidemiological conditions. Incorporating individual-level data on vaccination status and therapeutic interventions may also enhance its accuracy and relevance for ongoing and future use in diverse clinical settings.

A second limitation is that our study was conducted in high-complexity emergency care settings; thus, to use the model in lower-complexity healthcare settings, further validation may be required. Third, we did not include in this validation study other models developed for COVID-19 or for CAP, such as the CCEDRRN COVID-19 Mortality Score, the SEIMC score, and PSI, because some of them were not thoroughly developed and validated, or available, or usable in our context, given the need for laboratory assessments not readily available in our emergency settings (6).

Despite its clinical utility, the 4C model is not exempt from the risk of misclassification. When applying a low-risk threshold (≥4%) to guide hospitalization decision, some patients with low predicted mortality may be admitted unnecessarily. In these cases, clinical reassessment during hospitalization is essential to ensure timely discharge and avoid unnecessary use of healthcare resources. Conversely, patients classified as low risk (<4%) may still present atypical signs of severity not captured by the model or may deteriorate rapidly after emergency department discharge. Given the potential consequences of false-negative risk assessments, the 4C model should always be used alongside clinical judgment and appropriate follow-up to minimize decision-making errors and ensure patient safety.

In conclusion, the 4C Mortality Score outperformed NEWS2, CRB-65, CURB-65, and qSOFA in predicting 30-day mortality among patients presenting to the emergency department with suspected COVID-19. Given its ability to stratify patients into lower- and higher-risk-of-death categories, we recommend its use as a complementary tool to clinical judgment.

Integrating recalibrated 4C models into routine clinical practice may support more accurate triage and better resource allocation. However, it should not be used as a standalone criterion. Clinicians must remain aware of its limitations, apply it within a comprehensive and individualized clinical assessment, and ensure it is adapted to current clinical and epidemiological conditions through recalibration when necessary.

## Data Availability

The raw data supporting the conclusions of this article will be made available by the authors, without undue reservation.
