# Supplementary material for: Validation of CURB-65, CRB-65, NEWS2, qSOFA, and 4C scores for predicting mortality in COVID-19 patients across seven emergency Departments in Colombia
Source: Front Med (Lausanne). 2026 Feb 18;13:1738978. doi: 10.3389/fmed.2026.1738978 (PMC12956637; doi:10.3389/fmed.2026.1738978)
Supplement: Supplementary file 1 [file Supplementary_file_1.docx]

**SUPPLEMENTARY MATERIAL**

**Methods:**

**M1. Eligibility Criteria**

Inclusion criteria required patients with COVID-19, defined as confirmed SARS-CoV-2 infection along with at least one of the following symptoms: fever (temperature >38°C), newly onset cough (≤10 days), dyspnea, wheezing or crackles, asthenia or adynamia, odynophagia, anosmia or dysgeusia, headache, myalgia or arthralgia, fatigue or weakness, or acute diarrhea. SARS-CoV-2 infection was confirmed by either RT-PCR or antigen testing. Patients who were transferred to another institution within the first 24 hours of admission and those assessed via teleconsultation were excluded.

**M2. Prediction Models**

2.1 Table 1M. Original CURB-65 model.

| **Criterion** | **Points** |
| --- | --- |
| Confusion (new disorientation or altered mental status) | 1 |
| Urea >7 mmol/L (BUN >19 mg/dL) | 1 |
| Respiratory rate ≥30 breaths per minute | 1 |
| Blood pressure (SBP <90 mmHg or DBP ≤60 mmHg) | 1 |
| Age ≥65 years | 1 |

Adapted from Lim WS et al. *Thorax*. 2003;58(5):377–82. Available from: [www.thoraxjnl.com](http://www.thoraxjnl.com)

2.2 Table 2M. Interpretation of the CURB-65 model.

| **CURB-65 Score** | **Interpretation** | **Recommended Management** | **Mortality Rate** |
| --- | --- | --- | --- |
| 0 - 1 | Low risk | Likely suitable for home treatment. | **1.50%** |
| 2 | Intermediate risk | Consider hospital-supervised treatment (short stay inpatient or supervised outpatient care). | **9.20%** |
| ≥3 | High risk | Hospital management as severe pneumonia; assess for ICU admission, especially if CURB-65 score is 4 or 5. | **22%** |

Adapted from Lim WS et al. *Thorax*. 2003;58(5):377–82. Available from: [www.thoraxjnl.com](http://www.thoraxjnl.com)

2.3 Table 3M. Original NEWS2 model.

| **Parameter** | **3** | **2** | **1** | **0** | **1** | **2** | **3** |
| --- | --- | --- | --- | --- | --- | --- | --- |
| Respiration Rate (per min) | ≤8 | 9–11 | 12–20 | 21–24 | ≥25 | — | — |
| SpO₂ Scale 1 (%)^1^ | ≤91 | 92–93 | 94–95 | ≥96 | — | — | — |
| SpO₂ Scale 2 (%)^1^ | ≤83 | 84–85 | 86–87 | 88–92 | ≥93 on air | 93–94 on oxygen | 95–96 on oxygen |
| Air or Oxygen? | Oxygen | Air | — | — | — | — | — |
| Systolic Blood Pressure (mmHg) | ≤90 | 91–100 | 101–110 | 111–219 | ≥220 | — | — |
| Pulse (per-min) | ≤40 | 41–50 | 51–90 | 91–110 | 111–130 | ≥131 | — |
| Consciousness | — | — | Alert | CVPU^2^ | — | — | — |
| Temperature (°C) | ≤35.0 | 35.1–36.0 | 36.1–38.0 | 38.1–39.0 | ≥39.1 | — | — |

1. SpO₂ (Oxygen saturation) scoring in NEWS2 is based on two scales. Scale 1 is used for most patients. Scale 2 is applied only to patients with chronic hypercapnic respiratory failure (e.g., COPD with oxygen dependency). 2. CVPU: C = new confusion; V = response to voice; P = response to pain; U = unresponsive. CVPU is used to assess consciousness level in NEWS2.

Adapted from: Royal College of Physicians. *National Early Warning Score (NEWS) 2: Standardising the assessment of acute-illness severity in the NHS. Updated report of a working party*. London: RCP; 2017. Available from: <http://doi.wiley.com/10.1111/j.1478-5153.2012.00540_3.x>

2.4 Table 4M. Interpretation of the NEWS2 model.

| **Parameter** | **Clinical Risk** | **Response** | **30-day mortality (%)** |
| --- | --- | --- | --- |
| 0–4 | Low | Ward-based response.^1^ | 5.5 |
| Score of 3 in any parameter | Low–Medium | Urgent ward-based response.^2^ | 11.3 |
| 5–6 | Medium | Key threshold for urgent response.^3^ | 13.3 |
| 7 or more | High | Urgent or emergency response.^4^ | 27.6 |

1. Assessment by a competent registered nurse or equivalent, to decide change in frequency of clinical monitoring or escalation of care. 2. Urgent review by a ward-based doctor, to decide change in frequency of clinical monitoring or escalation of care. 3. Urgent review by a ward-based doctor or acute team nurse, to decide if critical care team assessment is needed. 4. Emergent assessment by a clinical team or critical care team and usually transfer to higher level of care. Adapted from: Royal College of Physicians. *National Early Warning Score (NEWS) 2: Standardising the assessment of acute-illness severity in the NHS. Updated report of a working party*. London: RCP; 2017. Available from: <http://doi.wiley.com/10.1111/j.1478-5153.2012.00540_3.x>

2.5 Table 5M. Original 4C model.

| **Variable** | **Points** |
| --- | --- |
| **Age (years)** |  |
| <50 | 0 |
| 50-59 | 2 |
| 60-69 | 4 |
| 70-79 | 6 |
| ≥80 | 7 |
| **Sex at birth** |  |
| Female | 0 |
| Male | 1 |
| **Number of comorbidities**^1^ |  |
| 0 | 0 |
| 1 | 1 |
| ≥2 | 2 |
| **Respiratory rate (breaths/min)** |  |
| <20 | 0 |
| 20-29 | 1 |
| ≥30 | 2 |
| **Peripheral oxygen saturation on room air (%)** |  |
| ≥92 | 0 |
| <92 | 2 |
| **Glasgow Coma Scale score** |  |
| 15 | 0 |
| <15 | 2 |
| **Urea (mmol/L)** |  |
| <7 | 0 |
| 7-14 | 1 |
| >14 | 3 |
| **C-reactive protein (mg/L)** |  |
| <50 | 0 |
| 50-99 | 1 |
| ≥100 | 2 |

1. Comorbidities were defined by using Charlson comorbidity index, with the addition of clinician defined obesity. Table adapted from Knight SR et al. *BMJ*. 2020;371:m4334. doi:10.1136/bmj.m4334.

2.6 Table 6M. Interpretation of the 4C Mortality Score model.

| **Risk Group** | **Derivation Cohort** | | **Validation Cohort** | |
| --- | --- | --- | --- | --- |
|  | **No. of Patients (%)** | **No. of Deaths (%)** | **No. of Patients (%)** | **No. of Deaths (%)** |
| Low (0-3) | 2574 (7.3%) | 45 (1.7%) | 1650 (7.4%) | 20 (1.2%) |
| Intermediate (4-8) | 8277 (23.3%) | 751 (9.1%) | 4889 (21.9%) | 486 (9.9%) |
| High (9-14) | 18,091 (51.0%) | 6310 (34.9%) | 11,664 (52.2%) | 3666 (31.4%) |
| Very High (≥15) | 6521 (18.4%) | 4320 (66.2%) | 4158 (18.6%) | 2557 (61.5%) |
| Overall | 35,463 | 11,426 | 22,361 | 6729 |

Adapted from supplementary material in Knight SR et al. *BMJ*. 2020;371:m4334. doi:10.1136/bmj.m4334. The expected mortality was calculated from the validation cohort.

2.7 Table 7M. Original qSOFA model.

| **Criteria** | **Score** |
| --- | --- |
| Respiratory rate ≥ 22 breaths per minute | 1 Point |
| Altered mental status (Glasgow Coma Scale < 15) | 1 Point |
| Systolic blood pressure ≤ 100 mm Hg | 1 Point |

Adapted from Seymour CW et al. *JAMA*. 2016;315(8):762–74. doi:10.1001/jama.2016.0288.

2.8 Table 8M. Interpretation of the qSOFA model.

| **qSOFA Score** | **Interpretation^1^** | | **30-day mortality^2^ (%)** |
| --- | --- | --- | --- |
| 0 | Low risk of sepsis-related mortality; no criteria met. | Not high risk for in-hospital mortality | 3% |
| 1 | Suggests possible sepsis, requires monitoring. |  |  |
| 2 | Increased risk of poor outcomes, consider early intervention. | High risk for in-hospital mortality (3- to 14-fold increase) | 24% |
| 3 | High risk of sepsis-related mortality, requires urgent evaluation and management. |  |  |

Adapted from Seymour CW et al. *JAMA*. 2016;315(8):762–74. doi:10.1001/jama.2016.0288. 1. The mortality percentage is adapted from the in-hospital mortality reported in the validation study by Freund et al., which included 1,081 patients presenting to an emergency department. Freund Y, Lemachatti N, Krastinova E, Van Laer M, Claessens YE, Avondo A, et al; French Society of Emergency Medicine Collaborators Group. Prognostic Accuracy of Sepsis-3 Criteria for In-Hospital Mortality Among Patients With Suspected Infection Presenting to the Emergency Department. JAMA. 2017 Jan 17;317(3):301-308. doi: 10.1001/jama.2016.20329.

**RESULTS**


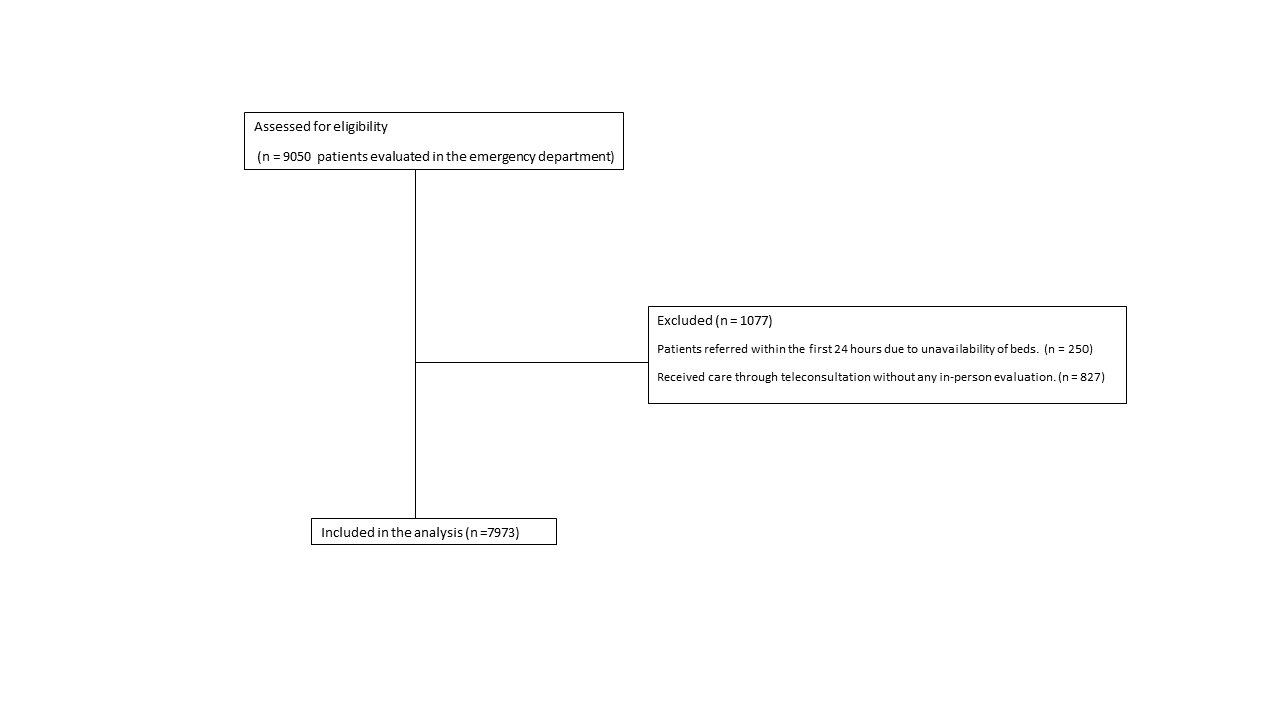


**F1. Figure 1.** Patients who presented to the emergency department from the Colombian cohort of the WHO Global Clinical Platform between March 2020 and September 2021, selected for the study based on eligibility criteria.


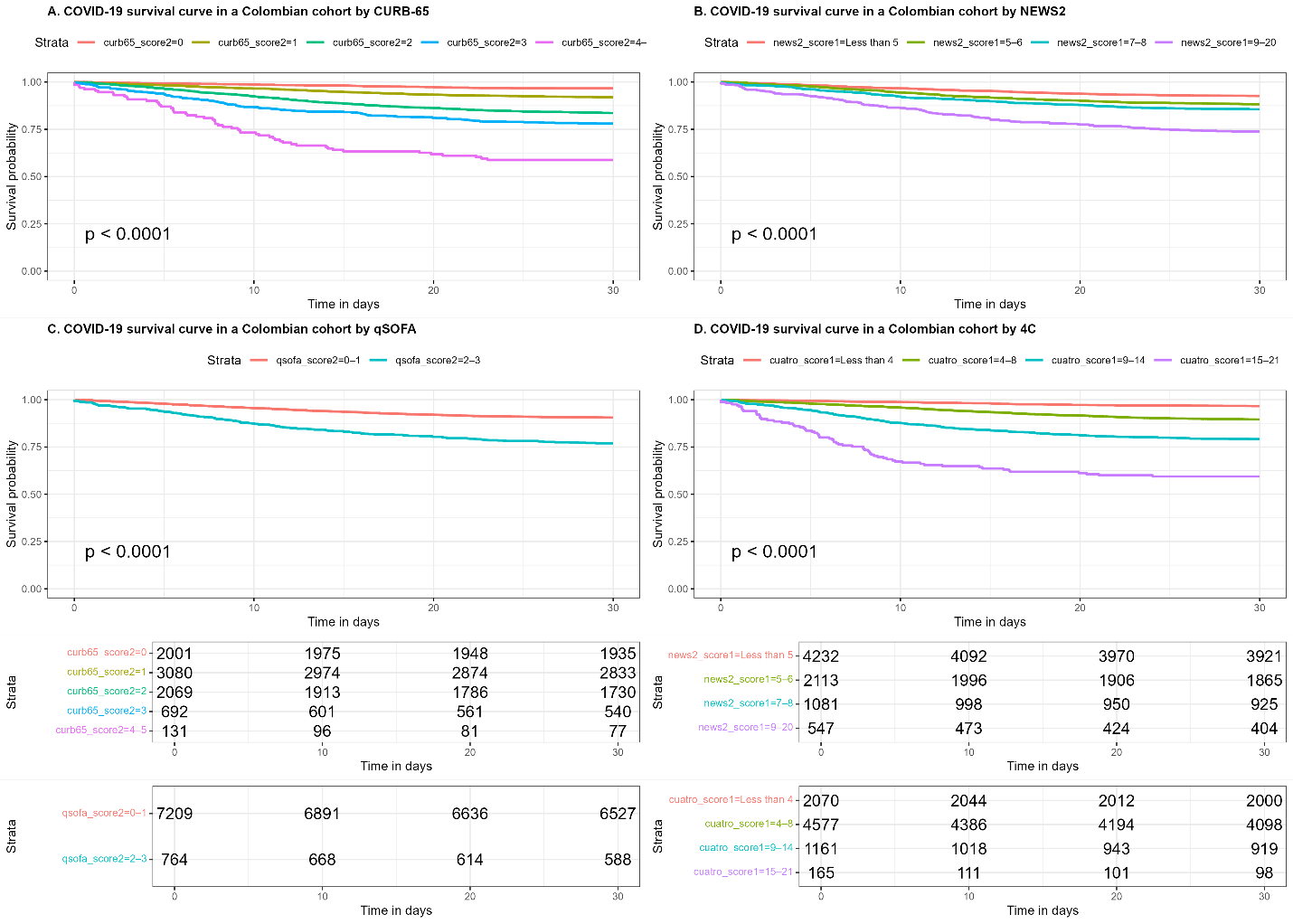


**F2. Figure 2.** Kaplan-Meier survival curves for a cohort of patients with SARS-CoV-2 infection presenting to seven high-complexity emergency departments in Colombia (March 2020 – September 2021), stratified by scores defined according to: (A) CURB-65, (B) NEWS2, (C) qSOFA, and (D) 4C. Log-rank test, p < 0.0001 for all models. NEWS2: National early warning score, qSOFA: quick Sequential [Sepsis-related] Organ Function Assessment, 4C: the Coronavirus Clinical Characterisation Consortium mortality score.

**
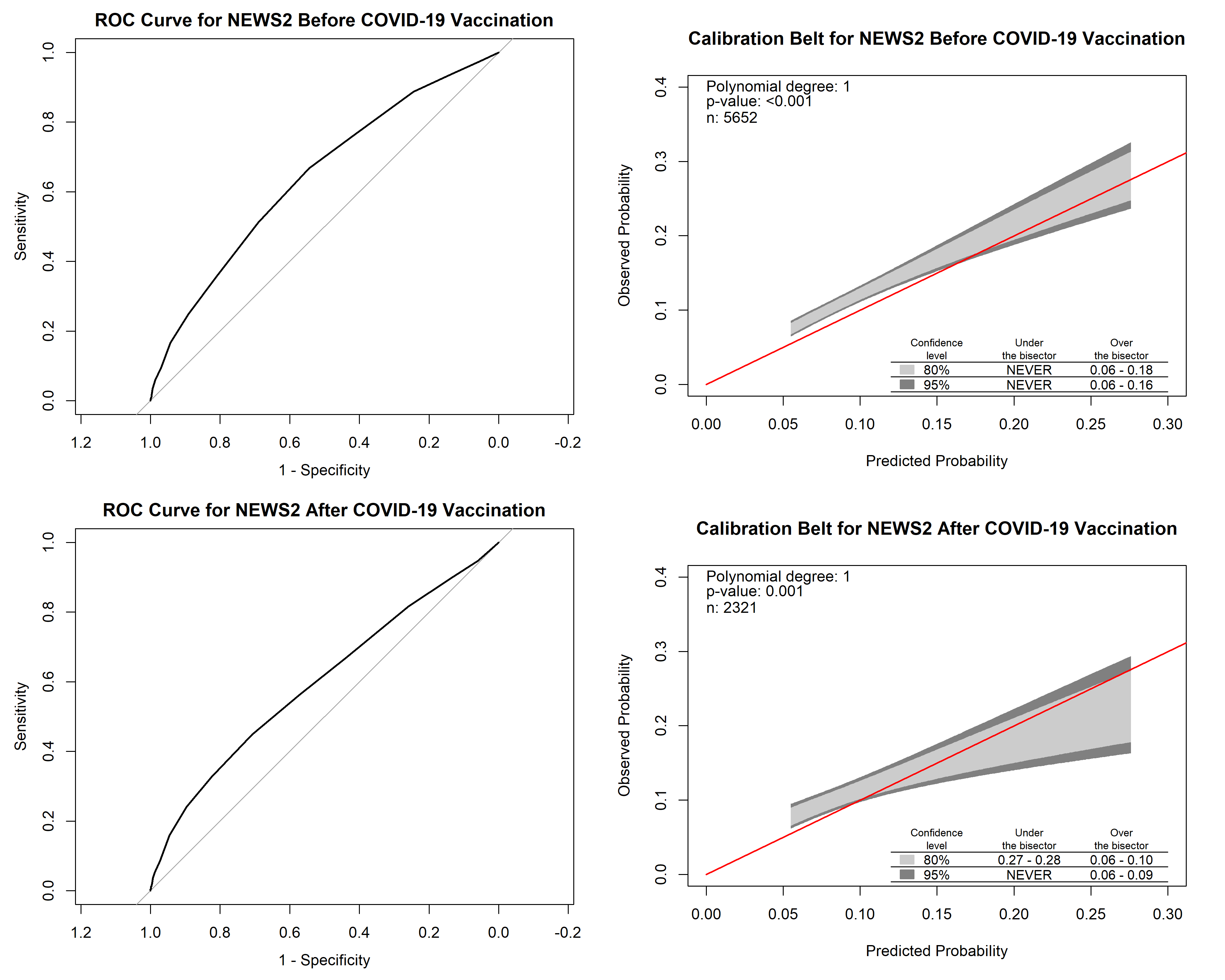
**

**F3. Figure 3.** Receiver operating characteristic (ROC) curves and calibration belts for the NEWS2 score to predict 30-day mortality after emergency department admission among adults with laboratory-confirmed SARS-CoV-2 infection treated in seven high-complexity emergency departments in Colombia, stratified by period before and after implementation of the national COVID-19 vaccination program.

1. ROC curve in the pre-vaccination period (March 2020–March 2021). Area under the curve (AUC) = 0.64 (95% CI, 0.62–0.67).
2. Calibration belt in the pre-vaccination period (March 2020–March 2021).
3. ROC curve in the post-vaccination period (April 2021–September 2021). AUC = 0.60 (95% CI, 0.56–0.64).
4. Calibration belt in the post-vaccination period (April 2021–September 2021).

**
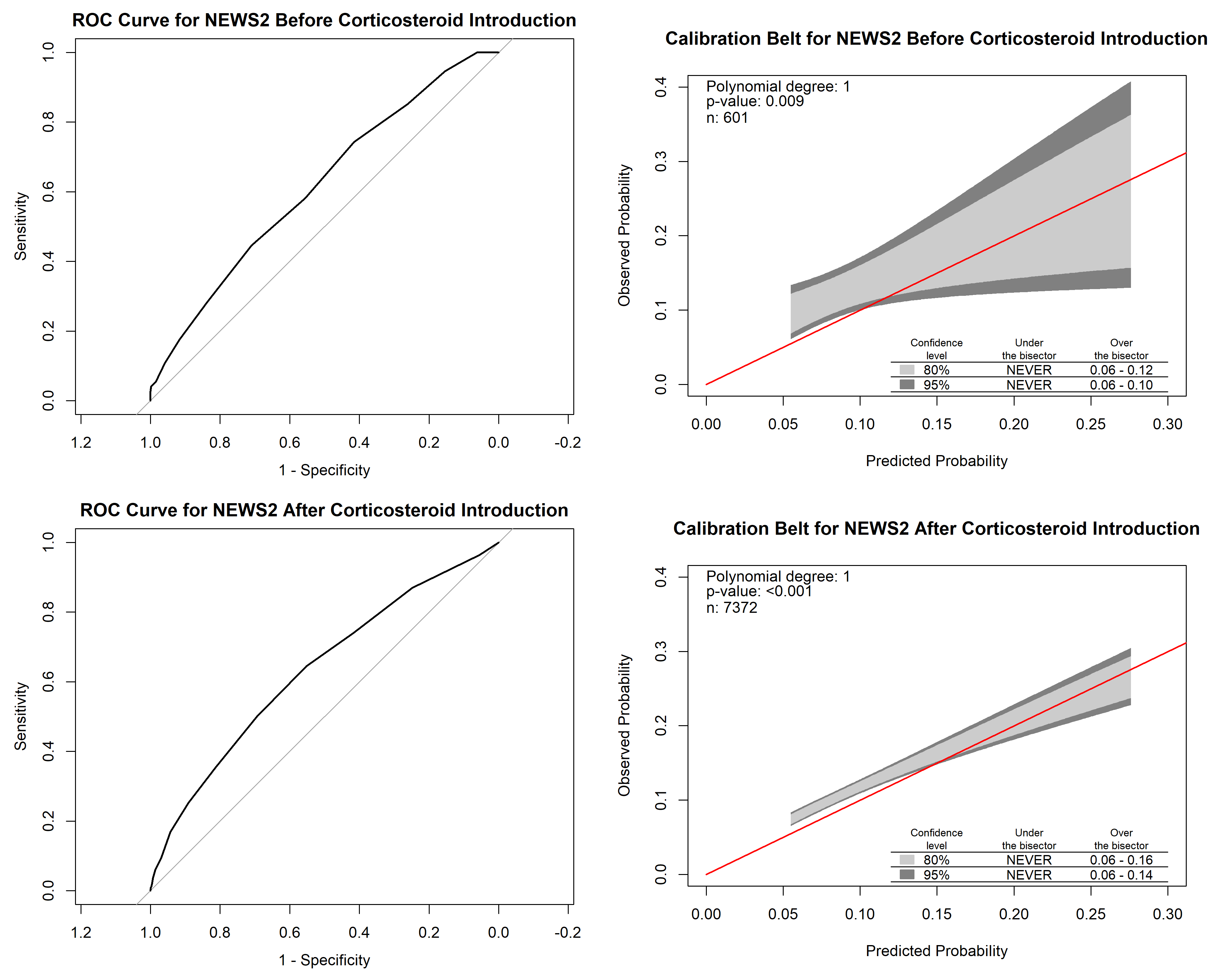
**

**F4. Figure 4.** Receiver operating characteristic (ROC) curves and calibration plots of the NEWS2 model for predicting 30-day mortality after emergency department admission in patients with SARS-CoV-2 infection from seven high-complexity emergency departments in Colombia, before and after the introduction of corticosteroid therapy.

**(A)** ROC curve before corticosteroid therapy implementation (March 2020–June 2020); AUC = 0.62 (95% CI: 0.65–0.68).

**(B)** ROC curve after corticosteroid therapy implementation (July 2020–September 2021); AUC = 0.63 (95% CI: 0.61–0.65).

**(C)** Calibration belt before corticosteroid therapy implementation (March 2020–June 2020).
**(D)** Calibration belt after corticosteroid therapy implementation (July 2020–September 2021).

**
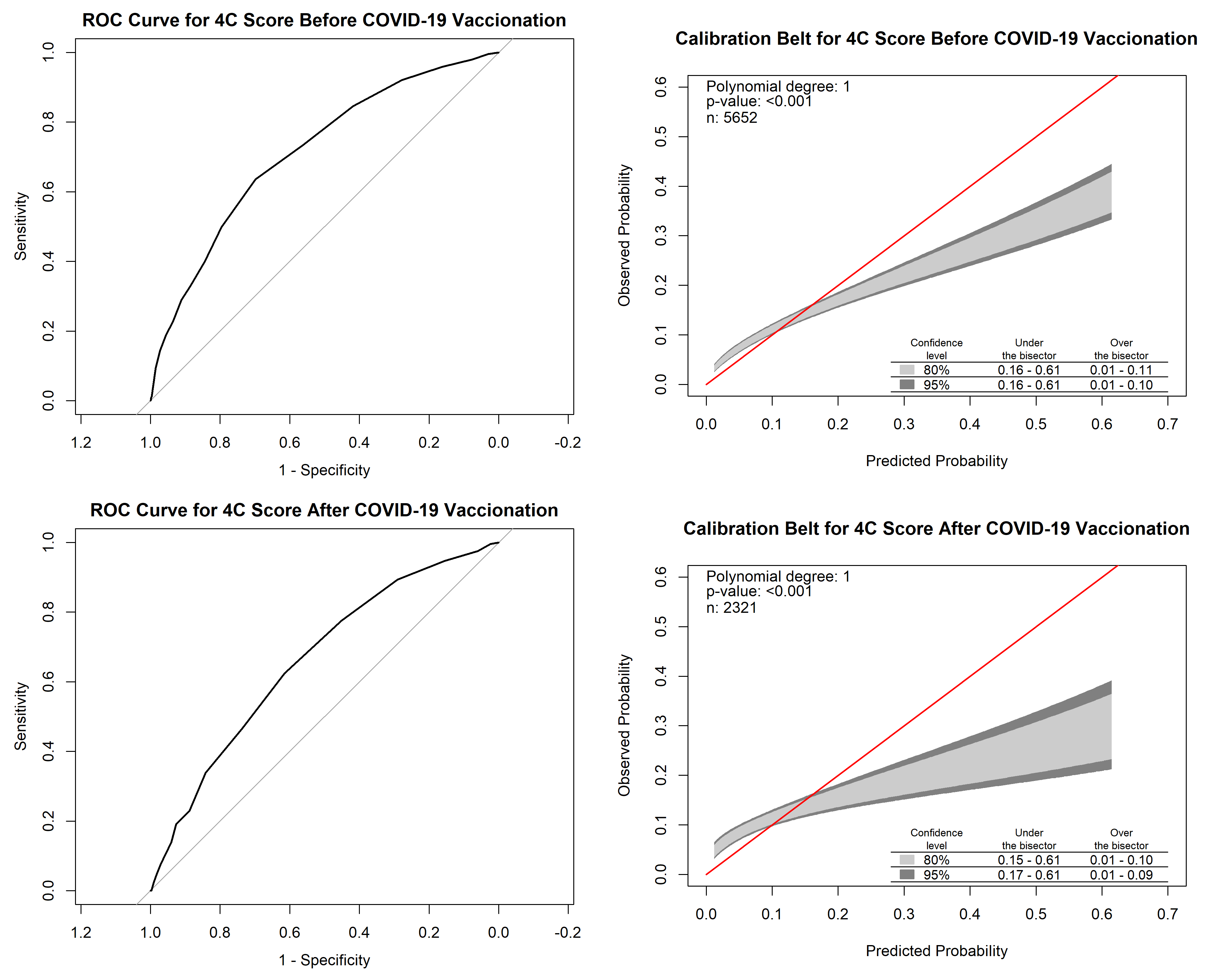
**

**F5. Figure 5.** Receiver operating characteristic (ROC) curves and calibration belts for the 4C mortality score to predict 30-day mortality after emergency department admission among adults with laboratory-confirmed SARS-CoV-2 infection treated in seven high-complexity emergency departments in Colombia, stratified by period before and after implementation of the national COVID-19 vaccination program.

(A) ROC curve in the pre-vaccination period (March 2020–March 2021). Area under the curve (AUC) = 0.72 (95% CI, 0.70–0.74).

(B) Calibration belt in the pre-vaccination period (March 2020–March 2021).

(C) ROC curve in the post-vaccination period (April 2021–September 2021). AUC = 0.70 (95% CI, 0.68–0.71).

(D) Calibration belt in the post-vaccination period (April 2021–September 2021).

**
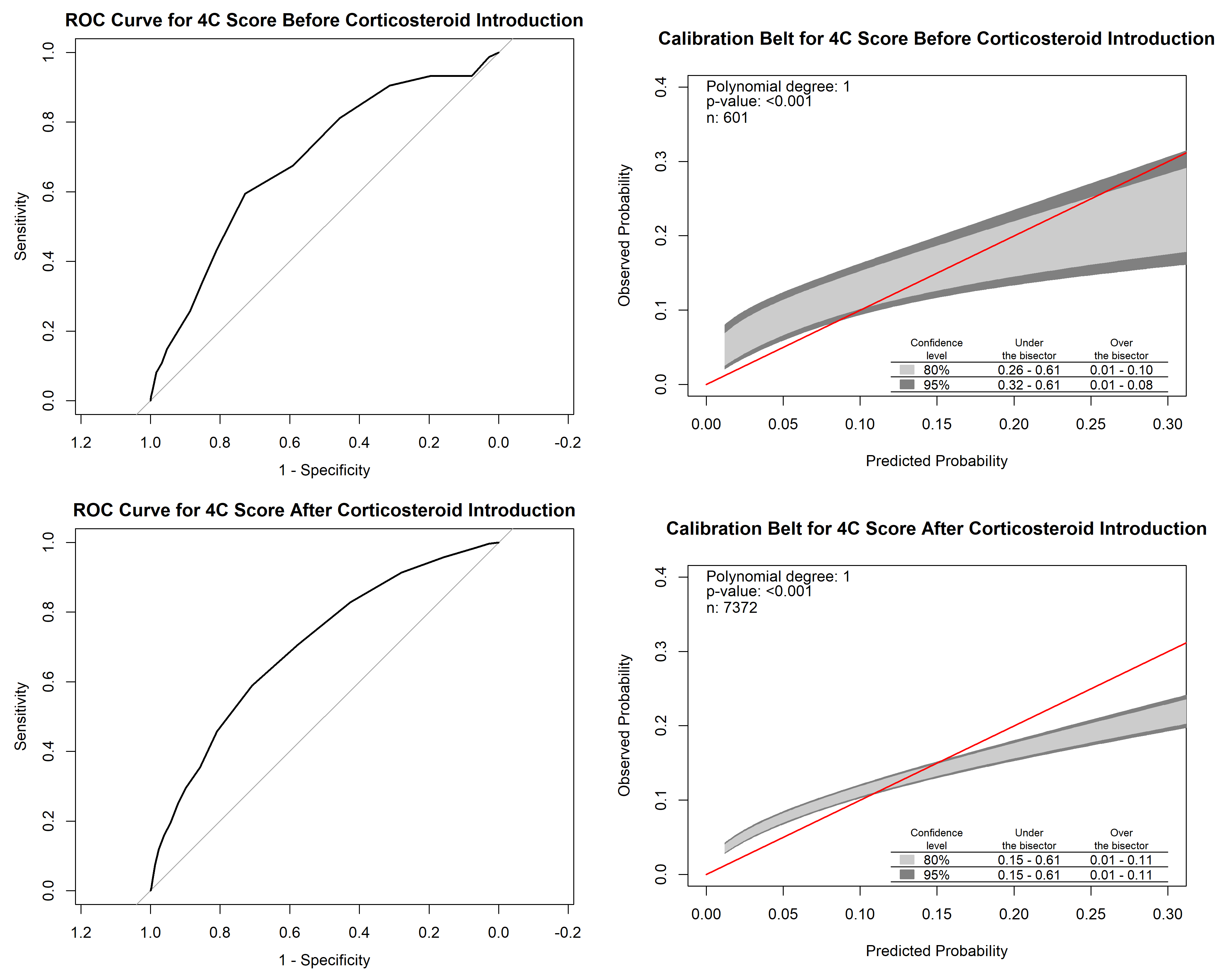
**

**F6. Figure 6.** Receiver operating characteristic (ROC) curves and calibration belts of the 4C mortality score for predicting 30-day mortality after emergency department admission among adults with SARS-CoV-2 infection treated in seven high-complexity emergency departments in Colombia, before and after implementation of corticosteroid therapy.

1. ROC curve in the pre-corticosteroid period (March 2020–June 2020); AUC = 0.70 (95% CI, 0.69–0.72).
2. ROC curve in the post-corticosteroid period (July 2020–September 2021); AUC = 0.70 (95% CI, 0.68–0.72).
3. Calibration belt in the pre-corticosteroid period (March 2020–June 2020).
4. Calibration belt in the post-corticosteroid period (July 2020–September 2021).

**
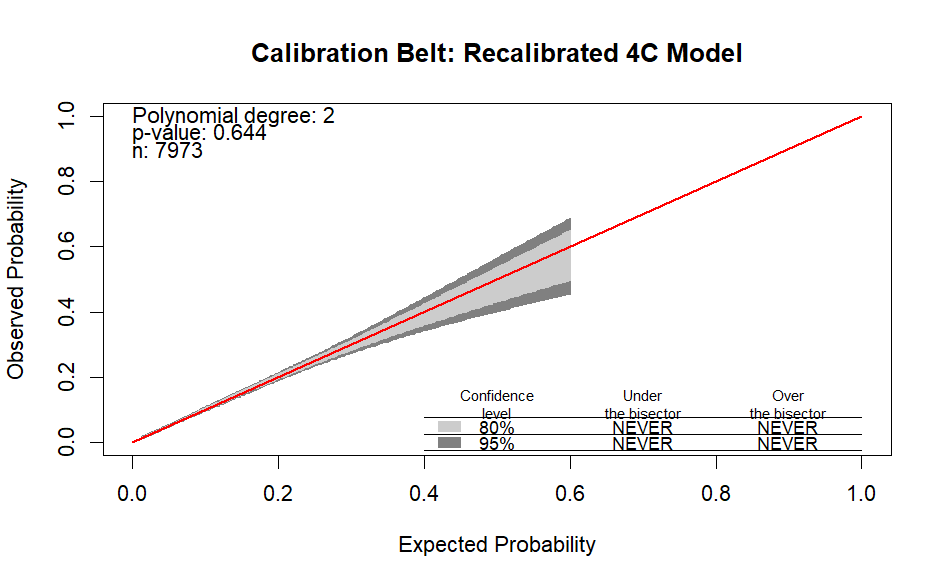
**

**F7. Figure 7.** Calibration belt of the recalibrated 4C mortality score for predicting 30-day mortality after emergency department admission in patients with SARS-CoV-2 infection across seven high-complexity emergency departments in Colombia (March 2020 – September 2021).


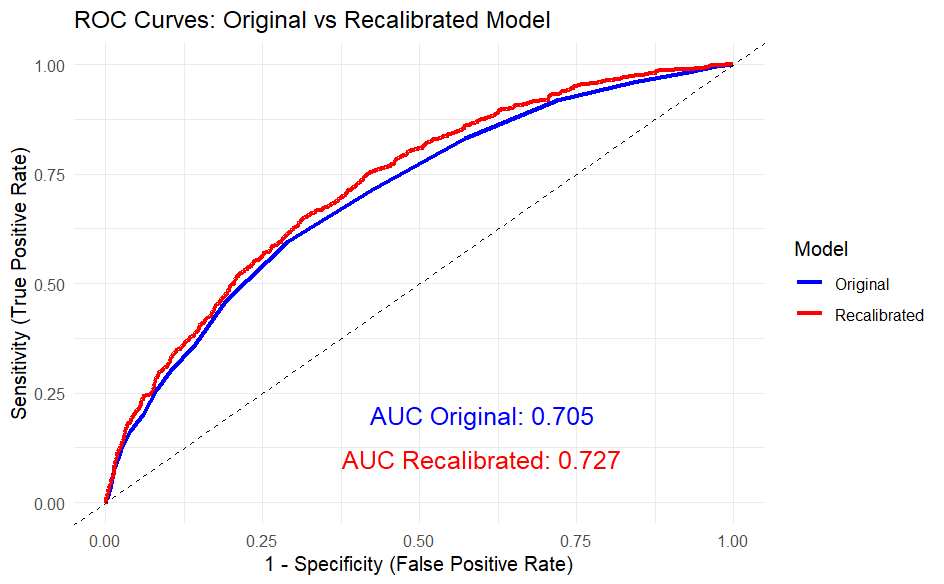


**F8. Figure 8.** Receiver operating characteristic (ROC) curves comparing the original and recalibrated 4C mortality scores for predicting 30-day mortality after emergency department admission in patients with SARS-CoV-2 infection across seven high-complexity emergency departments in Colombia (March 2020 – September 2021). 4C: Coronavirus Clinical Characterization Consortium mortality score.


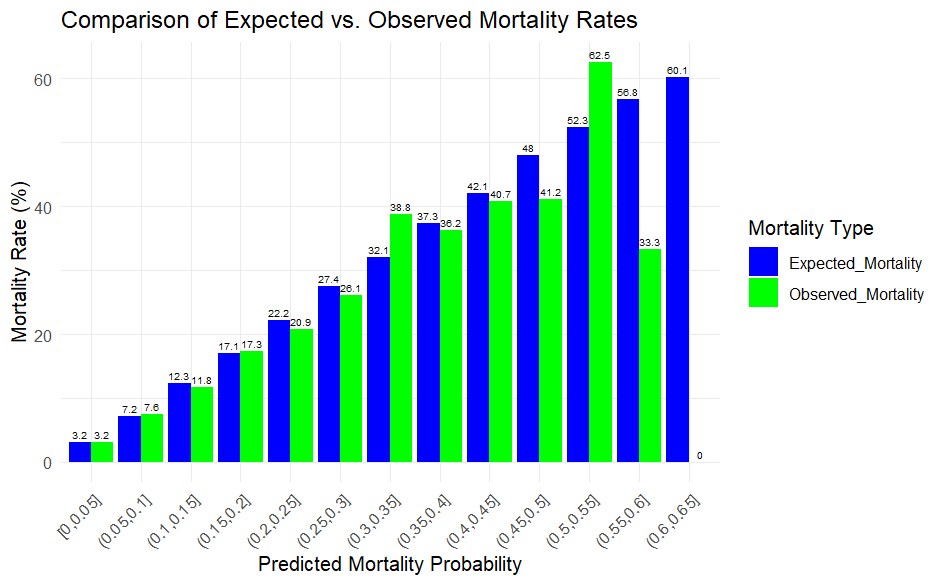


**F9. Figure 9.** Observed-to-expected event plots stratified by predefined risk groups for the recalibrated 4C mortality score in predicting 30-day mortality after emergency department admission in a cohort of patients with SARS-CoV-2 infection across seven high-complexity emergency departments in Colombia (March 2020 – September 2021).


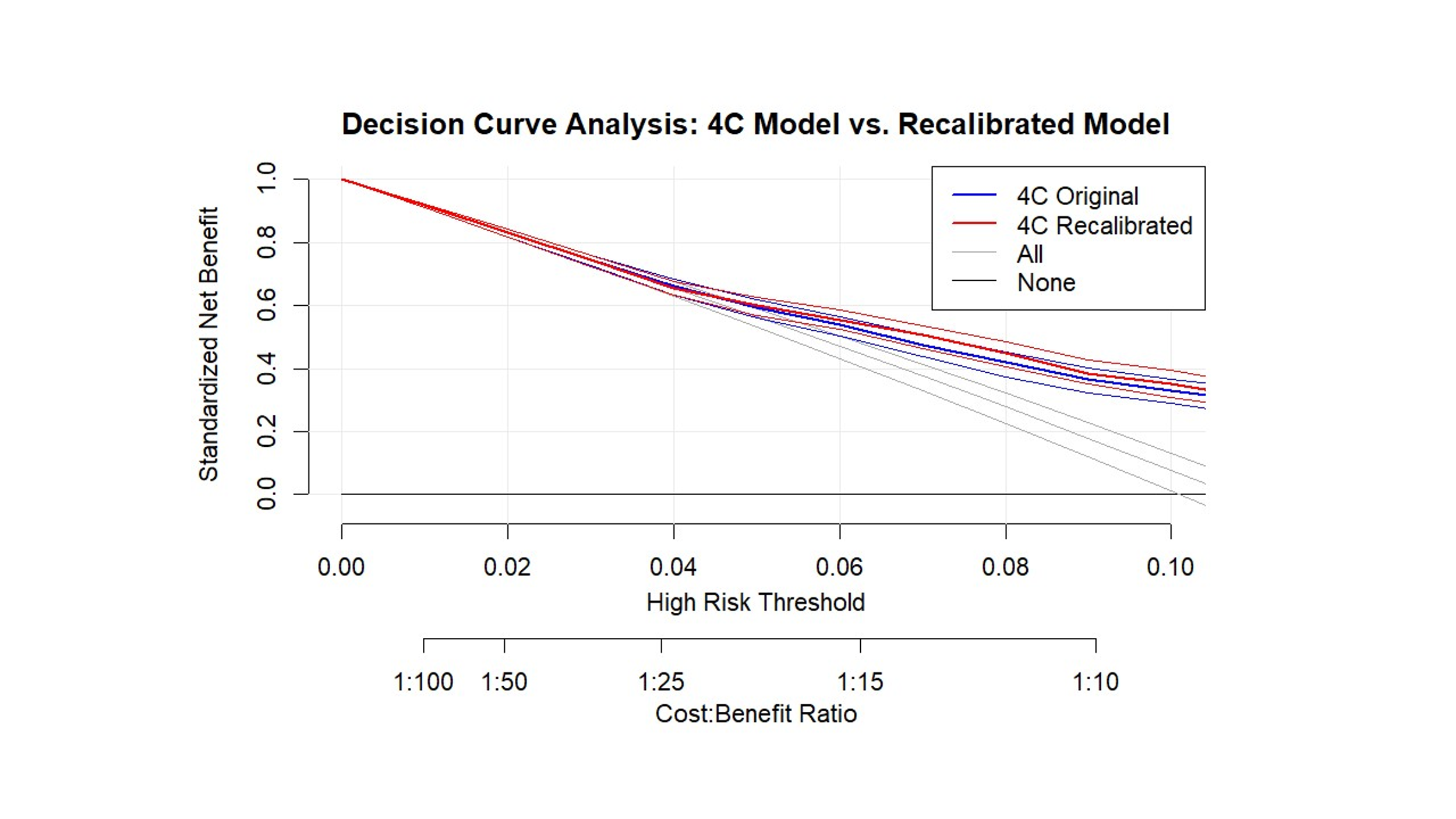


**F10. Figure 10**. Decision curve analysis comparing the 4C score and an all and none hospitalization approach in a cohort of patients with SARS-CoV-2 infection across seven high-complexity emergency departments in Colombia (March 2020 – September 2021). The x-axis represents the selected threshold and the corresponding cost-benefit ratio, while the y-axis displays the standardized net benefit.

**
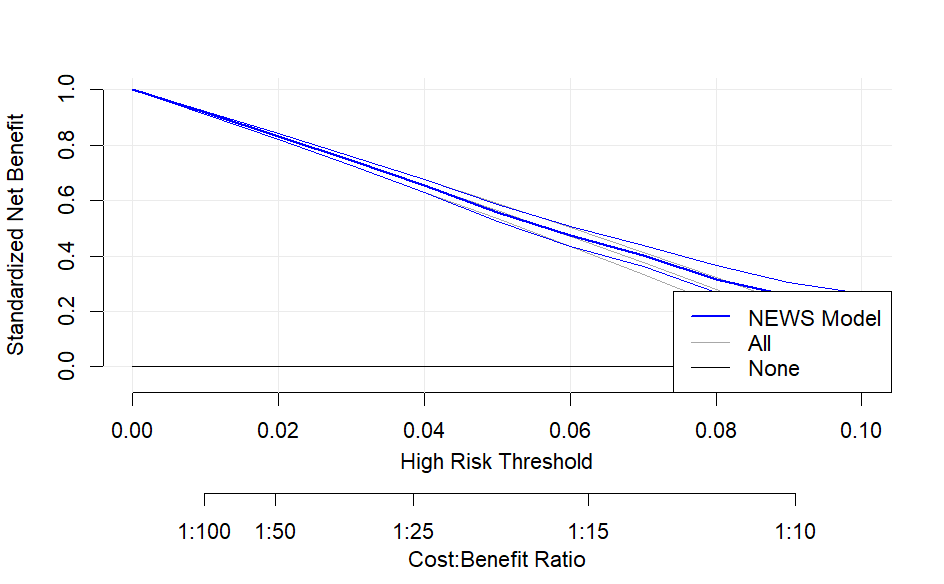
**

**F11. Figure 11**. Decision curve analysis comparing the NEWS2 score and an all and none hospitalization approach in a cohort of patients with SARS-CoV-2 infection across seven high-complexity emergency departments in Colombia (March 2020 – September 2021). The x-axis represents the selected threshold and the corresponding cost-benefit ratio, while the y-axis displays the standardized net benefit.

**T1R. Observed-to-expected ratio for predicting 30-day mortality following emergency department admission in a cohort of patients with SARS-CoV-2 infection across seven high-complexity emergency departments in Colombia (March 2020 – September 2021), stratified by predefined risk groups for CURB-65 (A1, B1), NEWS2 (A2, B2), qSOFA (A3, B3), and 4C (A4, C4).**

| **Score** | **N** | **%total** | **Expected (Predicted) events** | **Observed events** | **Expected (Predicted) risk (%)** | **Observed risk (%)** | **Ratio O/E^1^** |
| --- | --- | --- | --- | --- | --- | --- | --- |
| **A. CURB65** | | | | | | | |
| 0 | 2001 | 25.1 | 12 | 66 | 0.6 | 3.3 | 5.5 |
| 1 | 3080 | 38.6 | 83 | 246 | 2.7 | 8.0 | 3.0 |
| 2 | 2069 | 26.0 | 141 | 339 | 6.8 | 16.4 | 2.4 |
| 3 | 692 | 8.7 | 97 | 152 | 14 | 22.0 | 1.6 |
| 4 o 5 | 131 | 1.6 | 36 | 54 | 27.8 | 41.2 | 1.5 |
| **B. NEWS2** | | | | | | | |
| 0 - 4 | 4232 | 53.1 | 233 | 309.0 | 5.5 | 7.3 | 1.3 |
| 5 - 6 | 2113 | 26.5 | 239 | 247.0 | 11.3 | 11.7 | 1.0 |
| 7 - 8 | 1081 | 13.6 | 144 | 156 | 13.3 | 14.4 | 1.1 |
| >9 | 547 | 6.9 | 151 | 143 | 27.6 | 26.1 | 0.95 |
| **C. qSOFA** | | | | | | | |
| 0 - 1 | 7209 | 90.4 | 216 | 685.0 | 3.0 | 9.5 | 3.2 |
| 2 - 3 | 764 | 9.6 | 183 | 178.0 | 24.0 | 23.3 | 0.97 |
| **D. 4C Mortality score** | | | | | | | |
| 0 - 3 | 1866 | 24.0 | 19 | 63 | 1.2 | 3.4 | 2.8 |
| 4 - 8 | 4577 | 58.9 | 453 | 481 | 9.9 | 10.5 | 1.1 |
| 9 - 14 | 1161 | 14.9 | 365 | 241 | 31.4 | 20.8 | 0.7 |
| 15 - 21 | 165 | 2.1 | 101 | 67 | 61.5 | 40.6 | 0.7 |

1: Ratio observed/expected(predicted) events
